# Comparative Analysis of Tear Composition in Humans, Domestic Mammals, Reptiles, and Birds

**DOI:** 10.3389/fvets.2020.00283

**Published:** 2020-05-22

**Authors:** Ana Cláudia Raposo, Ricardo Dias Portela, Marcela Aldrovani, Thiago Doria Barral, Dayse Cury, Arianne Pontes Oriá

**Affiliations:** ^1^School of Veterinary Medicine and Zootechny, Federal University of Bahia, Salvador, Brazil; ^2^Institute of Health Sciences, Federal University of Bahia, Salvador, Brazil; ^3^Post-Graduation Program in Animal Science, Franca University, Franca, Brazil; ^4^Brazilian Institute of Ophthalmology and Blindness Prevention, Bahia School of Medicine and Public Health, Salvador, Brazil

**Keywords:** cholesterol, glucose, ocular surface, protein, tear composition, urea

## Abstract

Tears are an important component of the ocular surface protection mechanism and are in close contact with the corneal epithelium and the environment. Their composition is well-known in humans; however, there are few investigations on the composition and function of tears in reptiles, birds and others mammals, which would elucidate the mechanisms governing the maintenance of ocular homeostasis. In this work, electrophoretic profiles and an evaluation of total protein, albumin, urea, glucose, and cholesterol concentrations in tears of semi-aquatic, terrestrial, and marine reptiles (*Caiman latirostris, Chelonia mydas, Caretta caretta, Eretmochelys imbricata, Lepidochelys olivacea*, and *Chelonoidis carbonaria*), birds (*Tyto furcata, Rupornis magnirostris* and *Ara ararauna*), and mammals (*Equus caballus* and *Canis lupus familiaris*) were apresented. Human tear components and respective blood serum samples were used as references. The electrophoretic analysis revealed similarities whithin same Classes. The results of the tear–blood serum relationship and the comparison to human tear components showed particularities that are potentially derived from a homeostatic response to the environment. When the tear compositions of animals belonging to different ecological clusters were compared, marked differences were observed in total protein and urea concentrations. Thus, reptile, bird, and mammalian tears are complex fluids with differing concentrations of biochemical components that are potentially a result of the animals' adaptation to different environments.

## Introduction

The ecomorphological design of the vertebrate eye reflects the characteristics of the species' environment and lifestyle (1, 2) and although the morphological aspects of the eye are similar for all vertebrates, its microstructural and molecular features vary among species from different ecological niches (3–6). Evidence shows that the conformation, extension, and cell types of the tear apparatus vary among reptiles, birds, and mammals (7–9).

Tears are important components of the ocular surface, and are produced by different animals, from fish to mammals (6, 7, 10). They protect the eye from external damage and help maintain corneal and conjunctival health by preventing dryness and promoting tissue cooling (11–16). They can therefore be considered an interface between the environment and the ocular surface that is highly influenced by both of these two elements (4, 17, 18). Tears contain including lipids, carbohydrates, proteins, electrolytes, metabolites, and hormones (19, 20) and these compounds participate in the transport of other molecules, maintain the stability of the ocular surface, promote adherence and induce a protection mechanism against pathogens (16–18).

Different methods have been developed to study the tears of humans and experimental animal models, but very few authors have evaluated the composition of the tears of non-mammalian animals, although this evaluation can be easily performed using biochemical and electrophoretic approaches (12, 13, 21). To date, no study has compared tear composition among vertebrates belonging to different taxonomic ranks (Classes) or environments, and nothing has been documented about the composition of the tears produced by reptiles and birds.

Based on previous studies indicating that the environment has a direct influence on the ocular surface epithelium microprojections (4–6, 22), it is hypothesized that a similar influence can be exerted on tear composition. Thus, the composition of tear fluid was examined in six species of reptiles (*Caiman latirostris, Chelonia mydas, Caretta caretta, Eretmochelys imbricata, Lepidochelys olivacea*, and *Chelonoidis carbonaria*), three species of birds (*Tyto furcata, Rupornis magnirostris*, and *Ara ararauna*), and two domestic mammals (*Equus caballus* and *Canis lupus familiaris*). These findings were correlated to ecological and taxonomic indicators.

## Materials and Methods

### Species and Ethical Aspects

Human tear and serum samples were collected from 5 women and 5 men, aged 25–45 years, with normal values of tear production and no ocular surface abnormalities as shown by slit-lamp biomicroscopy (Kowa Company®, Torrance, CA). None of the patients included in the research had a prior history of neoplasms or systemic metabolic diseases, nor did they use systemic or topical medications that affect ocular homeostasis; none wore contact lenses. Protocols involving humans were approved by the Ethics Committee in Research of the Institute of Health Science, Federal University of Bahia (protocol number 2.388.777) and met the Brazilian legislation and ethical principles of the Helsinki Declaration. Written informed consent was obtained from all individuals.

The number of screened animals and the criteria for their selection and collection methods were based on previous studies in humans and other animals, which also described that no differences in the tear composition were observed when tears were collected using different methodologies (23, 24). Protocols for tear and blood serum sample collection from reptiles, birds, and non-human mammals were conducted as described by the Statement for the Use of Animals in Ophthalmic and Vision Research of the Association for Research in Vision and Ophthalmology, and were approved by the System of Authorization and Information on Biodiversity, Brazilian Ministry of Environment (SISBIO protocol numbers 27,489 and 50,054), and by the Ethics Committee on Animal Experimentation of the School of Veterinary Medicine and Zootechnology (protocol number 72/2016). All of the animals in this study were adults, male or female, kept under human care, with a balanced, and supervised diet (Table 1). A physical examination was performed before the ocular examination, and animals with any indications of systemic or ocular diseases were excluded from the study. The criteria used for species selection was the inclusion of mammalian, bird, and reptile species belonging to different ecological niches, and animals from whom it could be possible to collect the tear fluid samples. Furthermore, since each species produces different tear volumes (7, 15, 25, 26), a different number of animals of each species were screened with the objective to collect a total tear volume per species that would be sufficient to be used in all the analyzes herein proposed. As a consequence of the ethical aspects regarding wild animals and native fauna (as defined by the Brazilian Ministry of Environmental Issues) and the availability of species found in captivity that could be clinically evaluated, the number of species and animals sampled was limited.

**Table 1:** Description of reptiles, birds and mammals used for tear and blood serum collection.

**Class**	**Common name**	**Species and initials**	***n***	**Source**	**Habitat in captivity**	**Diet**	**Tear collection method**
Reptilia	Broad-snouted caiman	*Caiman latirostris*	35	Mister Cayman**—**commercial breeding	Semi-aquatic (terrestrial/ freshwater)	Chicken and meat (carnivore)	Schirmer strip
	Green turtle	*Chelonia mydas*	9	TAMAR Project	Aquatic (marine)	Fish, algae, and vegetables (omnivore)	Syringe
	Loggerhead turtle	*Caretta caretta*	10	TAMAR Project	Aquatic (marine)	Fish, algae, and vegetables (omnivore)	Syringe
	Hawksbill turtle	*Eretmochelys imbricata*	9	TAMAR Project	Aquatic (marine)	Fish, algae, and vegetables (omnivore)	Syringe
	Olive ridley turtle	*Lepidochelys olivacea*	5	TAMAR Project	Aquatic (marine)	Fish, algae, and vegetables (omnivore)	Syringe
	Red-footed tortoise	*Chelonoidis carbonaria*	12	Wild Animal Triage Center (CETAS)	Terrestrial (forested area)	Vegetables, feed, and meat, or chicken (omnivore)	Micropipette
Aves	Common barn owl	*Tyto furcata*	9	CETAS	Aerial (forested area)	Meat or slaughtered prey (mice) (carnivore)	Schirmer strip
	Roadside hawk	*Rupornis magnirostris*	6	CETAS	Aerial (forested area)	Meat and chicken or slaughtered prey (mice) (carnivore)	Schirmer strip
	Blue-and-yellow macaw	*Ara ararauna*	6	CETAS	Aerial (forested area)	Fruits, vegetables, feed and seeds (herbivore)	Schirmer strip
Mammalia	Dog	*Canis lupus familiaris*	8	Federal University of Bahia	Terrestrial (domestic conditions)	Commercial dog food (carnivore)	Schirmer strip
	Horse	*Equus caballus*	15	Military Police of Bahia	Terrestrial (confinement)	Commercial horse feed and hay (herbivore)	Microcapillary

### Tear and Blood Serum Collection

Tears were collected between February 2016 and September 2017 in the morning hours from municipalities in Northeast Brazil. All animals were restrained by physical techniques, and anesthetic eye drops were not used. All of the sampled animals had free access to food and were not fasted prior to the tear and blood sampling. Tears of *C. latirostris*, birds, dogs, and humans were collected using Schirmer tear strips (Ophthalmos®, São Paulo, Brazil) following the protocol described by Oriá et al. (25). All strips were from the same lot and were inserted in the ventral conjunctival sac and maintained in the fornix until the moistened portion reached 30 mm. Sea turtle tears were collected with a disposable syringe (3 mL; BD®, São Paulo, Brazil) due to their high viscosity (Video). *C. carbonaria* tears were collected with a micropipette, with the pipette tip inserted in the lower conjunctival sac; *E. caballus* tears were collected from the medial canthus of the eyelid with a microcapillary tube (26). The choice of the collection method was based on previous studies on humans and other animals that are taxonomically related to the animals herein studied (7, 25, 26), and considered the animal's well-being at the moment of sampling. The samples were stored at −20°C until further processing.

In this study, the blood serum evaluation was used to complement the clinical examination, and these data were related to the values of the biochemical components found in tears. Venous puncture was performed on the same day as tear collection. The collection sites were: the occipital venous sinus in *C. latirostris*, the jugular vein in chelonians, birds, dogs, and *E. caballus*, and the ulnar vein of humans. Blood serum samples were obtained by centrifugation for 10 min at 14,000 × *g* after clotting. Serum samples were stored at −20°C until further processing. Pools were made with the tears obtained from each species in an experimental approach similar to other studies (19, 23, 27), and considering the need of a high volume of sample from each species to perform all the analyzes proposed in this study.

### Tear Electrophoresis

The electrophoretic profile was obtained using the pooled tears from each species through a conventional one-dimensional SDS-PAGE protocol under denaturing conditions, according to Rebouças et al. (28). A volume of pooled tears from each species containing 50 μg total protein was applied to the gel. Molecular masses were determined by comparision to molecular weight standards (Kaleidoscope Prestained Standard® Bio-Rad, Hercules, CA). Protein bands were observed by Coomassie Brilliant Blue staining.

### Biochemical Analysis of Tears and Sera

After thawing, pooled tear samples and blood serum samples were used for the determination of biochemical compound concentrations. Total protein was quantified by the bicinchoninic acid method using a commercial kit (Thermo Scientific, Rockford, IL) and serum and tear albumin, urea, glucose, and cholesterol concentrations were also determined using commercially available kits (LabTest®, Belo Horizonte, Brazil) according to the manufacturer's recommendations. All of the evaluations were performed in duplicate and the results were expressed as means.

### Data Analyses

The biochemical components were evaluated using scatter diagrams of individual values and descriptive statistics. Associations of lacrimal composition with animal taxonomy and habitat were studied by density plots for sample distributions and statistically determined using Mood's non-parametric test, with significance set at *P* < 0.05. To establish levels of chemical similarity between tears of the different studied species, the values obtained for the different biochemical components were placed in a data matrix and submitted to Euclidean distance calculation. Cut-off distances, automatically calculated by statistical software, were used to define the formation of clusters, which were represented by dendrograms. Calculations were performed using Minitab 18 software (Minitab, San Diego, CA).

## Results

### Tear Electrophoretic Profiles

**Figure 1:**
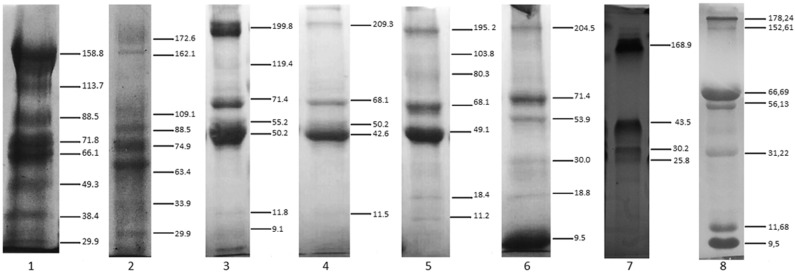
SDS-PAGE profile of tears from reptiles, birds and mammals. (1) *Caiman latirostris*, (2) *Chelonoides carbonaria*, (3) *Rupornis magnirostris*, (4) *Tyto furcata*, (5) *Ara ararauna*, (6) *Canis lupus familiaris*, (7) *Equus caballus*, (8) human. Staining with Coomassie Brilliant Blue. Numbers on the right indicate molecular mass values (kDa). Note the marked distinction in reptile protein bands, and similarities in the bands among birds, with a 50-kDa band seen for all of them. Among the mammals, a greatest similarity was demonstrated between the electrophoretic profiles of *Canis lupus familiaris* and humans.

The tear electrophoretic profile revealed protein bands with molecular masses ranging from 29 to 172 kDa for reptiles (*C. latirostris* and *C. carbonaria*), 9 to 209 kDa for birds, and 9 to 204 kDa for mammals (Figure 1). There were similarities within the same Class for birds and reptiles, and in mammals for dogs and humans. No clear distinction of protein bands for sea turtle tears was observed (Figure S1), which had the lowest protein concentration of the studied species. *C. latirostris* and *C. carbonaria* had the highest number of identified bands out of all studied species (*n* = 8). For birds, there was a high-intensity band with a molecular mass of ~50 kDa. For mammals, the species with the smallest number of bands was *E. caballus* (*n* = 4).

### Tear Biochemical Composition

**Figure 2:**
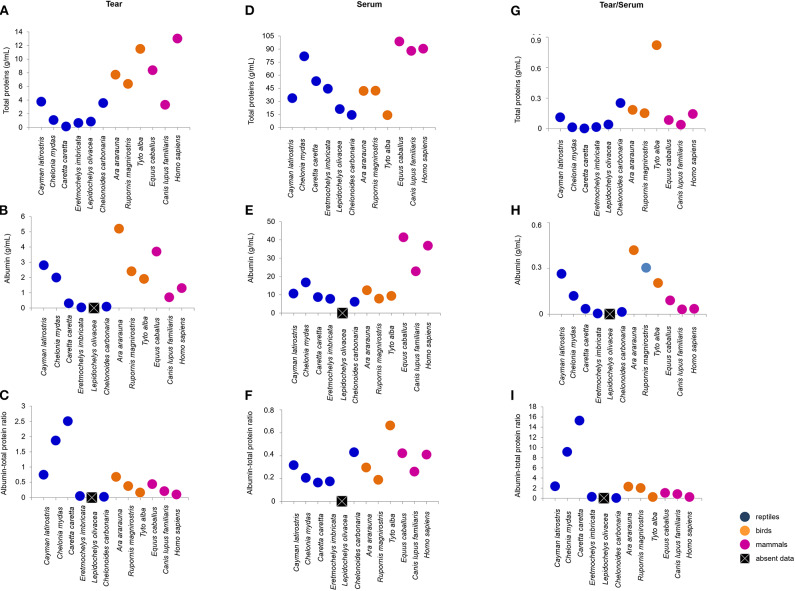
Total protein and albumin concentrations, and albumin-to-total protein ratio in tears and blood serum, and tear-to-blood serum concentration ratios for reptiles, birds and mammals. **(A–C)** shows respective tear component concentrations, **(D–F)** show respective blood serum component concentrations, and **(G–I)** show respective tear-to-blood serum concentration ratios. To check for hypothetical differences in composition for reptile, bird and mammalian tears, the animals were grouped by taxonomic Class. These results may serve as reference parameters for the evaluated species and provide information on tear components.

**Figure 3:**
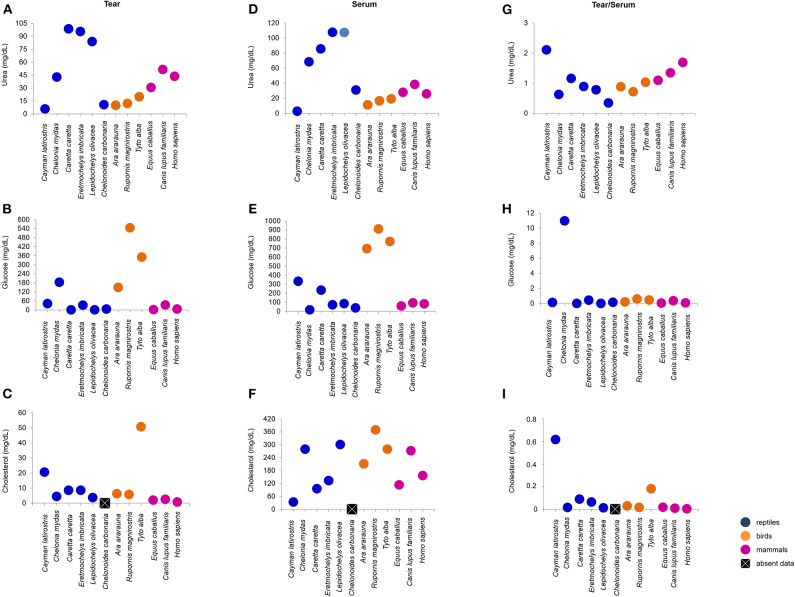
Urea, glucose and cholesterol concentrations in tears and blood serum, and tear-to-blood serum concentration ratios for reptiles, birds and mammals. **(A–C)** show respective tear component concentrations, **(D–F)** show respective blood serum component concentrations, and **(G–I)** show respective tear-to-blood serum concentration ratios. To check for hypothetical differences in composition for reptile, bird and mammalian tears, the animals were grouped by taxonomic Class. These results may serve as reference parameters for the evaluated species and provide information on tear components.

Tears of all Classes presented different concentrations of total protein, albumin, urea, glucose and cholesterol. *C. caretta* and *L. olivacea* did not present detectable of glucose, concetrations and in *C. carbonaria* and *L. olivacea*, it was not possible to quantify cholesterol or albumin, respectively, due to small sample size. Figures 2, 3 (Figure S2) shows a dispersion diagram of the concentrations obtained for each biochemical component evaluated, enabling visualization of their differences and similarities among species grouped into Classes.

Total protein concentration in the tears of birds and mammals was higher than for the four species of sea turtle, and human tears had the highest concentration of total protein. The most similar concentrations were found for the semi-aquatic and terrestrial reptiles (3.76 and 3.57 mg/dL, respectively). *C. mydas, C. caretta, E. imbricate*, and *L. olivacea* presented the lowest concentrations of total protein (Figure 2A). There were no similarities in albumin concentration among the Classes or ecological niches, with highest values for *T. furcata* (11.5 mg/dL), *C. latirostris* (2.8 mg/dL) and *E. caballus* (3.7 mg/dL), and lowest values for *E. imbricata* (0.03 mg/dL) and *C. carbonaria* (0.09 mg/dL) (Figure 2B). Due to the differences in albumin values, variation in the albumin-to-total protein concentration ratios was observed (Figure 2C).

Sea turtle tears had the highest concentration of urea, followed by mammals. In general, birds presented lower variation in the concentration of this metabolite (Figure 3A). For glucose, bird tears had the highest concentration, followed by *C. mydas* (183.28 mg/dL) (Figure 3B). Tear cholesterol concentrations were lowest among mammals and highest for *T. furcata* and *C. latirostris* (50.8 and 20.6 mg/dL, respectively) (Figure 3C).

### Biochemical Evaluation of Tears and Serum

The biochemical components of the blood sera were analyzed together with the tears' molecular composition. Serum total protein values were highest among mammals, and wide variation was found between the values obtained for birds and reptiles (Figure 2D). Serum albumin was similar between birds and reptiles (7.9 to 12.4 and 6.1 to 16.7 mg/dL, respectively), and the highest values were found for mammals (Figure 2E). The serum albumin-to-total protein concentration ratio was highest for *T. furcata* (0.66), *C. carbonaria* (0.43), *E. caballus* (0.42), and humans (0.4) (Figure 2F).

Serum urea concentrations were highest for sea turtles and lowest for *C. latirostris* (2.7 mg/dL). Among the birds, values ranged from 11.3 to 19.2 mg/dL and for mammals, 25.8 to 38.3 mg/dL (Figure 3D). The birds had the highest values of serum glucose, followed by *C. latirostris* and *C. caretta* (332.1 to 233.1 mg/dL, respectively). The lowest serum glucose concentrations were for *C. mydas* (16.7 mg/dL) and *C. carbonaria* (38 mg/dL) (Figure 3E). The values obtained for serum cholesterol differed among the studied species and groups, with the highest value for *R. magnirostris* and the lowest for *C. latirostris* (369 and 33.3 mg/dL, respectively) (Figure 3F).

The tear-to-blood serum ratios revealed similarities within the Classes for glucose (with the exception of *C. mydas*) and cholesterol (with the exception of *C. latirostris* and *T. furcata*) (Figures 3H,I). Among the compounds evaluated in the mammalian group, similarities for total protein, albumin, albumin-to-total protein ratio and cholesterol were found (Figures 2G–I, 3I). For birds, similarities were found among non-protein compounds (Figures 3G–I). No other evaluated parameters showed similar ratios within Classes.

### Differences in Biochemical Composition Between Human and Animal Tears

**Figure 4:**
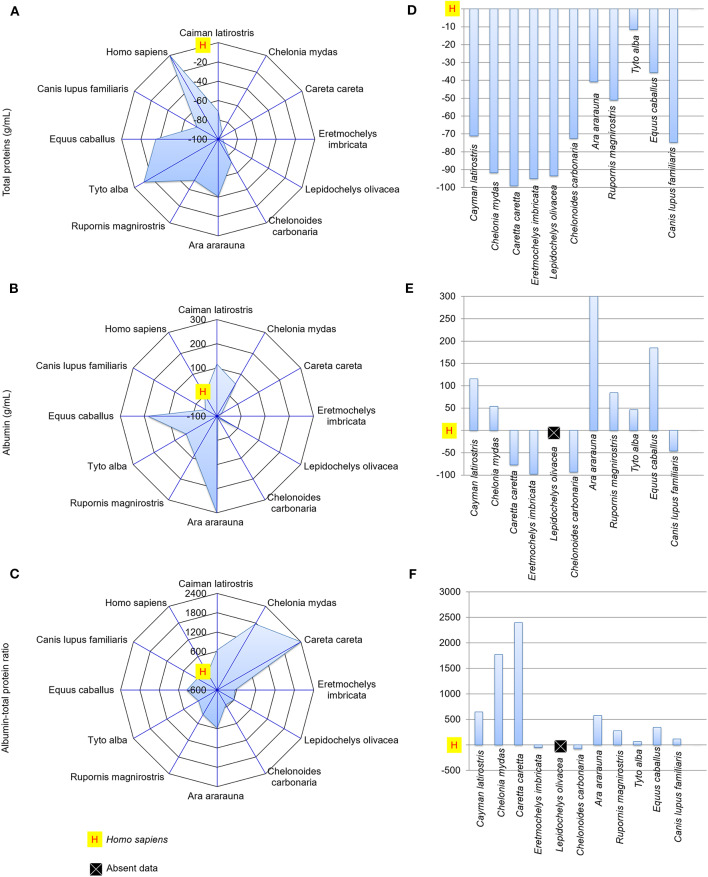
Total protein and albumin concentrations, and albumin-to-total protein concentration ratio in tears of reptiles, birds and mammals compared to human tears. Spider and bar graphs show the percentages obtained for total protein **(A,D)**, albumin **(B,E)**, and albumin-to-total protein concentration ratio **(C,F)** relative to human values. The spider graphs show the magnitude of the points' distances between the animals relative to humans for the different biochemical components. The bar graph compares the values obtained for the animals to the reference value (human), with an emphasis on the upper and lower values.

**Figure 5:**
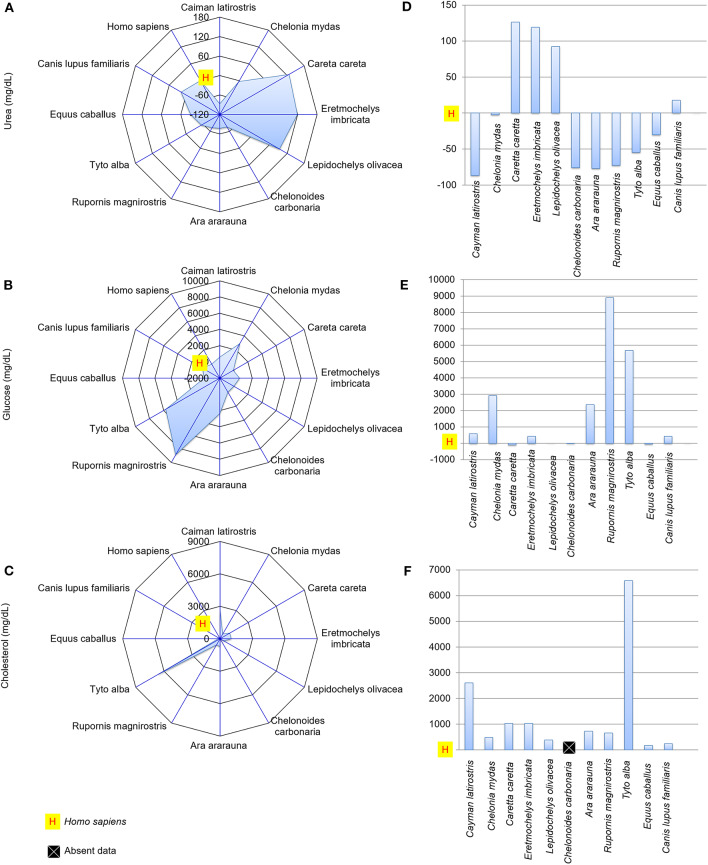
Urea, glucose and cholesterol concentrations in the tears of reptiles, birds and mammals compared to human tears. Spider and bar graphs show the percentages obtained for urea **(A,D)**, glucose **(B,E)**, and cholesterol **(C,F)** relative to human values. The spider graphs show the magnitude of the points' distances between the animals relative to humans for the different biochemical components. The bar graph compares the values obtained for the animals to the reference value (human), with an emphasis on the upper and lower values.

Figures 4, 5 presents spider and bar graphs showing the concentrations of the evaluated components in non-human species' tears relative to human tears. Tears of reptiles, birds, horse (*E. caballus*), and dog (*C. lupus familiaris*) had less total protein than human tears (Figures 4A,D). *E. caballus*, all birds, a species of sea turtle (*C. mydas*), and *C. latirostris* presented albumin-rich tears (Figures 4B,E).

The albumin-to-total protein concentration ratio in humans was higher than those for *E. imbricata* and *C. carbonaria* (Figures 4C,F). The urea concentrations in marine chelonian and dog tears were higher than in humans (Figures 5A,D). Most of the studied species presented higher glucose values than those found in human tears, except for *C. caretta, L. olivacea, C. carbonaria* and *E. caballus* (Figures 5B,E). All animals had higher cholesterol concentrations in their tears when compared to humans (Figures 5C,F). The spider graphs also showed cluster formation for albumin-to-total protein ratio, urea, and glucose, due to the similarities between relative values found for some species of the same taxonomic Class, particularly reptiles and birds.

### Associations of Tear Biochemical Composition With Taxonomy and Habitats

Associations of tear biochemical composition with taxonomy (reptiles, birds, and mammals) and habitat (semi-aquatic freshwater, marine, aerial, and terrestrial) were calculated using Mood's non-parametric test, which can be applied to small populations and detects differences in dispersion between populations that are identical in all other respects. The statistical analysis showed that taxonomic Class does not influence the content of any of the studied components in reptile, bird, or mammalian tears (Table 2), and animal habitat was significantly associated to the amount of total protein and urea in tears (Table 3).

**Table 2:** Comparison of biochemical parameters in the tears of reptiles, birds and mammals.

**Parameter**	**Reptile**		**Bird**		**Mammal**				
	**Median**	**Q3–Q1**	**Median**	**Q3–Q1**	**Median**	**Q3–Q1**	**Chi-Square**	**DF**	***P*-value**
Total protein (g/mL)	1.0	3.1	7.7	5.1	8.4	9.7	6.00	2	0.05
Albumin (g/mL)	0.3	2.3	2.4	3.3	1.3	3.0	0.78	2	0.676
Albumin-to-total protein ratio	0.7	2.1	0.3	0.5	0.2	0.3	0.78	2	0.676
Urea (mg/dL)	63.3	87.2	11.9	9.8	43.7	20.9	4.00	2	0.135
Glucose (mg/dL)	19.0	77.0	351.0	397.0	6.0	30.0	4.00	2	0.135
Cholesterol (mg/dL)	8.6	10.5	6.2	45.0	2.1	1.8	3.47	2	0.176

**Table 3:** Comparison of biochemical parameters in the tears of semi-aquatic, marine, aerial, and terrestrial species.

**Parameter**	**Semi-aquatic (freshwater)**		**Marine**		**Aerial**		**Terrestrial**				
	**Median**	**Q3–Q1**	**Median**	**Q3–Q1**	**Median**	**Q3–Q1**	**Median**	**Q3–Q1**	**Chi-Square**	**DF**	***P*-value**
Total protein (g/mL)	3.7	—	0.7	0.8	6.0	8.5	7.7	5.1	6.97	2	0.031^*^
Albumin (g/mL)	2.8	—	0.3	1.9	1.0	2.8	2.4	3.3	1.32	2	0.517
Albumin-to-total protein ratio	0.7	—	1.8	2.4	0.1	0.3	0.3	0.5	1.32	2	0.517
Urea (mg/dL)	5.7	—	89.8	45.1	37.1	34.0	11.9	9.8	6.97	2	0.031^*^
Glucose (mg/dL)	40.9	—	16.0	145.0	6.0	22.0	351.0	397.0	4.95	2	0.084
Cholesterol (mg/dL)	20.5	—	6.5	4.8	2.1	1.8	6.2	45.0	3.06	2	0.217

* *Indicates significant statistical association to the habitat, as defined by the Mood's non-parametric test (P < 0.05)*.

### Degree of Similarity Between Tears of the Different Species Studied as Revealed by Cluster Analysis of Their Biochemical Components

**Figure 6:**
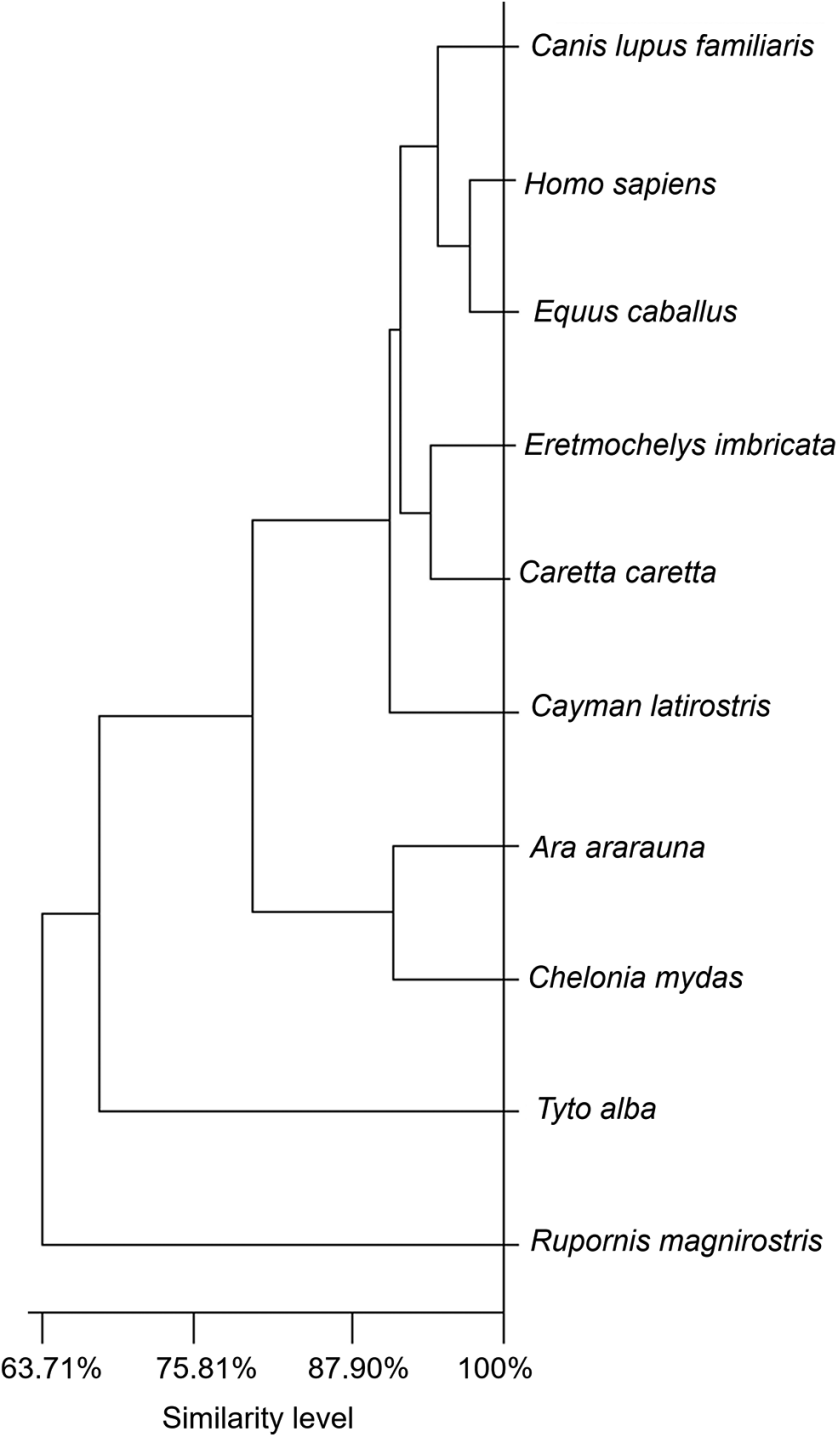
Dendrogram for evaluating similarities in the biochemical components of tears of reptiles, birds and mammals. The dendrogram shows the Euclidean distance obtained from the values of the biochemical components (total protein, albumin, albumin-to-total protein ratio, urea, glucose and cholesterol). Similarities were not directly correlated to taxonomic Class. The illustration shows the tears' different levels of similarity among the evaluated species.

A dendrogram based on Euclidian distance calculations (Figure 6) showed that the tears of birds of prey (*R. magnirostris* and *T. furcata*) present a less similar distributional balance of biochemical components compared to the tears of the other species (levels of similarity of 63.71% for *R. magnirostris* and 68.27% for *T. furcata*). In reptiles, a high compositional similarity existed between the tears of *C. caretta* and *E. imbricata* (94.27%). In the mammalian group, horse tears' biochemical composition was better correlated to that of human tears (similarity of 97.34%) than that of dog tears.

## Discussion

Vertebrates are distributed among aerial, terrestrial, and aquatic environments, and tears are in direct contact with these surroundings, protecting the eye from external conditions (7). In most of these animals, this is the organism's most exposed fluid, and it is a potentially major target of environment-induced modifications. Previous evaluations of the lacrimal glands have shown histological specificities among species, such as the presence of lymphocytic tissue in the Harderian gland of crocodilians and the absence of meibomian glands in the tarsal region in owls (7, 29) All reptiles, birds and mammals have mucus, lipids and water in their tears (7). However, this is the first study describing the composition of reptile and bird tears and providing a comparative analysis with mammalian tears.

An important aspect of tear-composition analysis is that it can reveal biomarkers for ocular and systemic diseases. Although the collection methods differed among the species in this study, the collection site was the same for all animals (lower conjunctival sac), to minimize possible effects on the results. It should be noted that some prior studies (23, 25, 27) did not observe any differences in the eletrophoretic profiles and crystalization of tears collected by Schirmer's strip or microcapillary tube.

Tear proteins are responsible for osmotic balance maintenance, defense and metabolite transport, and their characteristics have been described primarily in humans (20, 30). Previous studies on humans tears have reported the presence of mucoproteins and glycoproteins that promote stability of the ocular surface (17, 19, 30, 31). This finding may justify the high protein concentration found in the tears of *C. latirostris* compared to the other reptiles, because these animals are in close contact with a hypotonic aquatic freshwater environment and present a wide blink interval (5, 22, 32), both situations demanding highly stable tears.

Sea turtle tears had a lower amount of total protein than other reptilian species, as also seen by the SDS-PAGE profile. This lower protein concentration can be derived from epithelium modifications originating from the need to balance the environment's high salinity (4, 5), which can cause denaturation or even conformational changes of some proteins, such as those studied in humans tears. *T. furcata* also had a high protein concentration in its tears compared to other bird species, and this situation has to be considered together with the fact that these animals have a widely exposed ocular surface and reduced tear production (22, 29, 33); tear fluid with concentrated protein can present reduced evaporation via lipolytic substances carried by proteins or lipoproteins (17, 20, 30, 34). The viscous characteristic of the sea turtle tear is an interesting aspect and further proteomics and glycomics studies are being conducted with the objective to better understand this situation.

In this study, although tear protein concentration did not seem to be associated with animal taxonomy, the evaluation of tear-to-serum concentration ratios revealed that, except for birds, taxonomically close species might demonstrate a direct association between the biochemical compositions of these two fluids. This result can be attributed to species-specific metabolism or to ecological niches. Similarities were found in the electrophoretic profiles of taxonomically close species or those from the same environment. High-molecular-mass proteins (150–200 kDa) were found in all species and, in previous studies with dogs and humans (30, 31, 34, 35), these bands corresponded to glycoproteins (mucins), which maintain close contact with the corneal epithelium and provide tear stability.

The electrophoretic profiles of the tears of humans and other mammals are similar (24, 36), notably between humans and dogs. In contrast, heterogeneity was found between the profiles of human and non-mammalian tears, suggesting the occurrence of Class-specific peculiarities, or protein polymorphisms that could not be detected by the methodology used here. This hypothesis needs to confirmed by amino acid sequencing, which could reveal new molecular mechanisms associated with, for example, stability and adhesion of the tear to the cornea.

Albumin has a role in metabolite and ion transport in the serum, and in the maintenance of osmolarity in tears (20, 30); its molecular mass in human tears is ~69 kDa (37). Bands with similar molecular masses were found in all evaluated species, except for sea turtles, perhaps because of the low amount of protein in the latter's tears. In all species, a low tear-to-serum concentration ratio of albumin was found, similar to that described for humans (34). The high concentration of albumin found in the tears of birds, particularly *R. magnirostris* and *A. araruana*, compared to other species, could be the consequence of a mechanism that reduces impact at the air–ocular surface interface through the maintenance of tear osmolarity. In *E. caballus*, an animal adapted to long and fast runs, a high concentration of albumin in the tears was noted and, as previously described, in the serum (38).

Urea is a product of the metabolism of nitrogen compounds and its concentration range is influenced by food habits, or by specific production by the lacrimal gland (11, 20, 39, 40). In humans, who present the unrestricted passage of urea through the blood–tear barrier in the lacrimal gland, this metabolite is present at similar concentrations in tears and serum (11, 41), similar to what was observed for birds in this study. In sea turtles, high values of lacrimal urea were observed, which can be attributed to their salt gland (a modified lacrimal gland) that, similar to the kidney, excretes catabolites (40). Moreover, among the mechanisms that help maintain osmotic pressure balance on the ocular surface in marine animals, intensive transport of electrolytes (22), and urea contributes to this process. The urea concentration in tears of *C. latirostris* was widely different from its serum concentration, a situation strongly related to the fact that uric acid is the main excreted nitrogen compound in this reptile (42). Thus, it was not posible to find urea at high concentrations in *C. laritostris* serum samples; however, the elevated presence of urea in its tears might be the result of a biological adaptation driven by the effects of freshwater osmotic pressure on the ocular surface, because as already noted, urea is an important component in the osmotic balance on the ocular surface (11).

At first glance, the presence of glucose in the tear fluid might seem strange, representing a waste of energy. However, it must be remembered that the lacrimal fluid is highly important for ocular surface protection. A previous evaluation of tears as a non-invasive biomarker fluid for glycemia failed to establish a direct correlation between the physiological profile and tear glucose concentrations (43, 44). In the present study, the results showed higher glucose concentrations in bird tears and sera; these animals have been previously reported to have higher serum glucose concentrations than animals of other Classes (45). The high concentrations of glucose in *R. magnirostris* and *T. furcata* may also be associated with the uninterrupted gluconeogenesis that occurs, independent of fasting, in carnivorous birds (45). Glucose was not found in the tears of *C. caretta* or *L. olivacea*, two phylogenetically close species. A high concentration of glucose was observed in the tears of *C. mydas*, but not in its serum, likely an effect of factors such as food intake in captivity, fasting time prior to material collection, or even restraint stress (45, 46). Among the components studied, existed lower interspecies variation in the tear-to-serum concentration ratio of glucose, suggesting correlated glucose concentration in these two fluids for all species in this study, except *C. mydas*.

Cholesterol, a lipid that can be widely found in tears, is related to lubrication, structural maintenance, thermoregulation and bactericidal activity (20, 46, 47). The tears of the studied animals had more cholesterol than human tears, reasserting the importance of this molecule for reptiles, birds and domestic animals (47, 48). For humans, this component is produced by the meibomian gland; it is not directly derived from the serum. For *T. furcata*, the high concentration of tear cholesterol cannot be attributed to the collection method, since the species does not have lipid-producing glands in the eyelid region (29, 33). Except for *C. latirostris* and *T. furcata*, the tear-to-serum concentration ratios of cholesterol were similar among animals; thus it can be implied that cholesterol is more important for these two species, since both exhibit a long interval between eyelid incursions, resulting in lengthy exposure of the ocular surface.

The tears of birds of prey presented biochemical profiles with greater Euclidian distance, or less similarity, from the other species. The similarity between the biochemical profiles of the other species was 88.42%. This finding, in addition to the associations observed for animal habitat, suggest that species-specific particularities in lacrimal composition do not reflect a linear trajectory or history in animal evolution. The cluster variability and exceptions observed in this study apparently do not correspond to the positioning of the animals, or different Classes of vertebrates, on the taxonomic scale. Moreover, making evolutionary assumptions based on biochemical parameter reference values for tears and sera of vertebrate animals is difficult, because these parameters are profoundly affected by a variety of factors, such as changes in dietary habit, and interference in the quantitative expression of lipids, proteins and nitrogenous products as these are present in all body fluids, especially in reptiles and birds. In this work, strict procedures and criteria for animal inclusion were rigorously maintained, with the aim of obtaining more reproducible and homogeneous results, but it must be assumed that all of the cited factors might cause interference bias.

In conclusion, we suggest that variations in the tear composition of reptiles, birds and mammals are potentially associated with the type of environment in which the animal lives, and there are marked differences between tears from humans and tears from these animals. A more precise study of these associations, through proteomics, glycomics and metabolomics analyses, may aid in the development of new therapeutics for human and veterinary medicine. Moreover, this study provides information that can serve as a basis for further analyses using animals in experimentally controlled environments.

## Data Availability Statement

The raw data supporting the conclusions of this article will be made available by the authors, without undue reservation, to any qualified researcher.

## Ethics Statement

The studies involving human participants were reviewed and approved by Ethics Committee in Research of the Institute of Health Science, Federal University of Bahia. The patients/participants provided their written informed consent to participate in this study. The animal study was reviewed and approved by Ethics Committee on Animal Experimentation of the School of Veterinary Medicine and Zootechnology. Written informed consent was obtained from the owners for the participation of their animals in this study.

## Author Contributions

AO, MA, and RP conceived and designed the study. AR, TB, and DC carried out investigation and methodology. The data validation was made by AO, RP, and MA. All authors have read and accepted the manuscript as it is presented to the journal.

## Conflict of Interest

The authors declare that the research was conducted in the absence of any commercial or financial relationships that could be construed as a potential conflict of interest.
