# Supplementary material for: Comparative Analysis of Tear Composition in Humans, Domestic Mammals, Reptiles, and Birds
Source: Front Vet Sci. 2020 May 22;7:283. doi: 10.3389/fvets.2020.00283 (PMC7256680; doi:10.3389/fvets.2020.00283)
Supplement: Supplementary file 2 [file Data_Sheet_1.DOCX]

**SUPPLEMENTARY MATERIAL**

**S1 VIDEO.** Sea turtle (*Caretta caretta*) tear production**.** Note the high tear viscosity that blocks the capillary process in Schirmer strips, thus requiring a different means of tear collection. The only possible and available option was collection by syringe (MP4).

**S1 FIGURE.** SDS-PAGE profile of pooled tears from sea turtles**.** (1) *Caretta caretta*, (2) *Eretmochelys imbricata*, (3) *Lepidochelys olivacea*, (4) *Chelonia mydas*, (5) molecular weight standard. Staining with Coomassie Brilliant Blue. Note scarce distinction of protein bands for sea turtle tears (TIF).

**S2 FIGURE.** Biochemical compound concentrations in tear and blood serum samples from reptiles, birds and mammals**.** These values may serve as reference parameters for the evaluated species and provide information on tear components (TIF).
