# Supplementary figures and images for: Comparative Analysis of Tear Composition in Humans, Domestic Mammals, Reptiles, and Birds
Source: Front Vet Sci. 2020 May 22;7:283. doi: 10.3389/fvets.2020.00283 (PMC7256680; doi:10.3389/fvets.2020.00283)

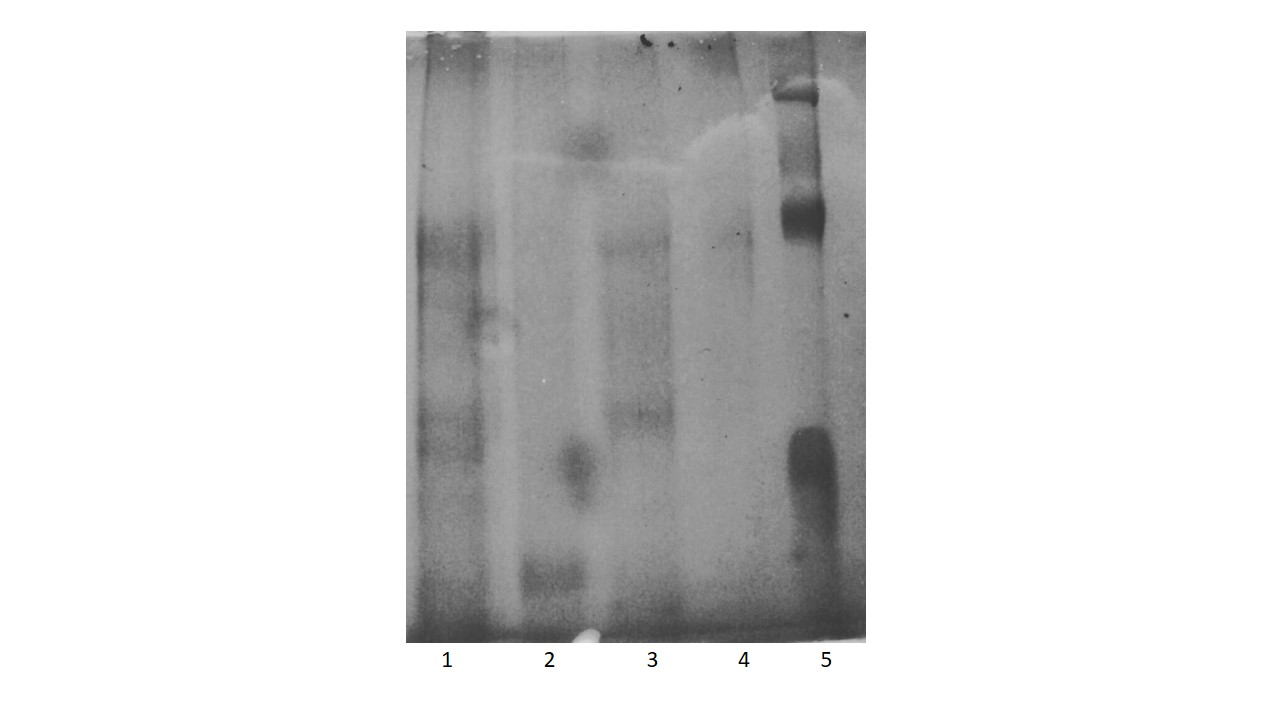

Supplement: Supplementary file 3 [file Image_1.TIF]

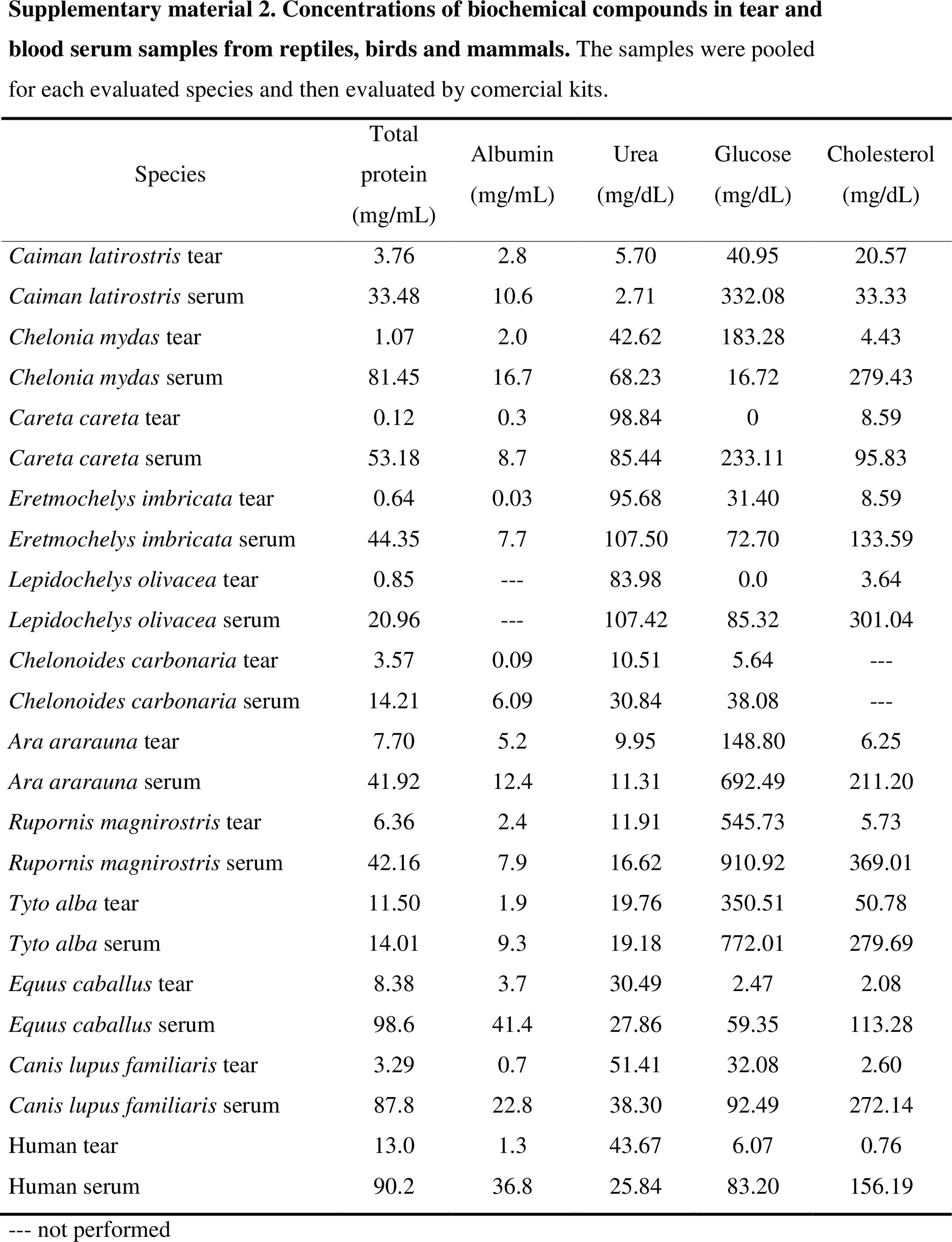

Supplement: Supplementary file 4 [file Image_2.TIF]
